# Non-coding RNA in osteoarthritis: mechanistic insights and future perspectives

**DOI:** 10.3389/fendo.2026.1851589

**Published:** 2026-07-14

**Authors:** Manali Jain, Anamika Kumari Anuja, Soniya Yadav, Jasmin Ara, Anukool Ojha, Chandra Prakash Chaturvedi

**Affiliations:** 1 Stem Cell Research Center, Department of Hematology, Sanjay Gandhi Post-Graduate Institute of Medical Sciences, Lucknow, India; 2 Department of Immunology, Sanjay Gandhi Post-Graduate Institute of Medical Sciences, Lucknow, India

**Keywords:** circRNAs, lncRNAs, miRNAs, non-coding RNA, osteoarthritis, signaling pathway

## Abstract

Non-coding RNAs (ncRNAs) have emerged as critical regulators of osteoarthritis (OA) pathogenesis by modulating gene expression at the epigenetic, transcriptional, and post-transcriptional levels. Among the major ncRNA classes, microRNAs (miRNAs), long non-coding RNAs (lncRNAs), and circular RNAs (circRNAs) regulate key biological processes involved in OA progression, including extracellular matrix degradation, synovial inflammation, chondrocyte apoptosis, autophagy, osteoclastogenesis, and subchondral bone remodeling. Increasing evidence demonstrates that ncRNAs influence multiple OA-associated signaling pathways, particularly NF-κB, TGF-β/SMAD, Wnt/β-catenin, PI3K/Akt, and MAPK/ERK, thereby contributing to cartilage degeneration and the failure of joint homeostasis. This review comprehensively summarizes the current understanding of ncRNA-mediated mechanisms in OA, with an emphasis on mechanistic validation, biomarker development, and therapeutic translation. Among all ncRNA classes, miRNAs currently possess the strongest experimental and translational evidence. miR-140-5p is the most extensively validated cartilage-protective miRNA and suppresses ADAMTS5-mediated extracellular matrix degradation in human OA cartilage, whole-body knockout mouse models, and *in vitro* systems. Additional miRNAs, including miR-146a, miR-21, and miR-34a, regulate inflammatory signaling, osteoclastogenesis, and chondrocyte survival by modulating NF-κB and TGF-β-related pathways. In contrast, many lncRNAs and circRNAs, including HOTAIR, MALAT1, NEAT1, and ciRS-7, remain predominantly supported by transcriptomic profiling and cell-based studies, with comparatively limited validation in primary human OA tissues. The review further highlights the emerging diagnostic potential of circulating ncRNAs detectable in serum, plasma, and synovial fluid as minimally invasive biomarkers for OA. However, current biomarker studies remain constrained by methodological heterogeneity, small cohort sizes, and a lack of longitudinal validation. In addition, recent advances in ncRNA-based therapeutics, including miRNA mimics, antagomiRs, antisense oligonucleotides, siRNA platforms, nanoparticle carriers, and exosome-mediated delivery systems, are critically discussed. Although these approaches demonstrate encouraging cartilage-protective and anti-inflammatory effects in preclinical OA models, substantial challenges related to delivery efficiency, tissue specificity, long-term safety, and clinical translation remain unresolved. Overall, ncRNAs represent promising molecular regulators and potential therapeutic targets that may contribute to future precision medicine strategies for managing OA.

## 1 Introduction

Osteoarthritis (OA) is the most prevalent degenerative joint disease worldwide and a major cause of chronic pain and disability (1, 2). Once considered a cartilage-only disorder, OA is now recognized as a complex whole-joint disease characterized by progressive cartilage degradation, synovial inflammation, osteophyte formation, and subchondral bone remodeling (3, 4). Mechanical stress, aging, obesity, metabolic dysfunction, genetic susceptibility, and chronic low-grade inflammation collectively contribute to disease initiation and progression (1, 4). Despite its high global burden, current OA management remains largely symptomatic, underscoring the need for a better understanding of the molecular mechanisms underlying disease pathogenesis (2, 5).

ncRNAs have emerged as important regulators of gene expression and cellular homeostasis (6–8). Although only a small fraction of the human genome encodes proteins, the majority of transcribed sequences consist of ncRNAs with diverse regulatory functions (6). Among them, microRNAs (miRNAs), long non-coding RNAs (lncRNAs), and circular RNAs (circRNAs) are the most extensively studied in skeletal biology and disease (9). These molecules regulate multiple biological processes, including inflammation, apoptosis, extracellular matrix remodeling, osteogenic differentiation, and signal transduction through pathways such as NF-κB, Wnt/β-catenin, PI3K/Akt, MAPK, and TGF-β signaling (9, 10).

Increasing evidence indicates that ncRNAs play central roles in OA pathogenesis by modulating chondrocyte function, synovial inflammation, extracellular matrix degradation, autophagy, and osteochondral remodeling (4, 11–13). Among the most extensively studied candidates, miR-140-5p, miR-146a, miR-21, and miR-34a have been shown to play pivotal roles in cartilage biology (14–16). These microRNAs are well-characterized regulators of cartilage homeostasis and inflammatory signaling (17–20). In contrast, many lncRNAs and circRNAs, including HOTAIR, MALAT1, NEAT1, and ciRS-7, remain supported primarily by transcriptomic and cell-based studies, with limited validation in primary human OA tissues (21–25). The stability and detectability of circulating ncRNAs in serum, plasma, and synovial fluid have also generated considerable interest in their potential as minimally invasive biomarkers and therapeutic targets for OA (1, 5).

This review recapitulates the current understanding of miRNAs, lncRNAs, and circRNAs in OA pathogenesis, emphasizing their mechanistic roles, biomarker potential, and emerging therapeutic applications. In addition, the review critically evaluates current translational challenges and highlights future directions for developing ncRNA-based precision diagnostics and therapies for OA.

## 2 Non-coding RNA biology and classification

The human genome is pervasively transcribed; however, only about 2% of the transcribed genome encodes proteins, and most transcripts are ncRNAs (6). Once considered transcriptional by-products or “junk” RNA, ncRNAs are now recognized as critical regulators of gene expression and cellular homeostasis. They participate in diverse biological processes, including chromatin remodeling, transcriptional regulation, RNA processing, post-transcriptional modification, signal transduction, and cellular differentiation (7, 8). Among the various classes of ncRNAs, the miRNAs, lncRNAs, and circRNAs are the most extensively studied in the context of bone health and disease.

MicroRNAs (miRNAs) are small, endogenous, single-stranded RNAs, approximately 20–24 nucleotides long, that negatively regulate gene expression by binding primarily to the 3′ untranslated regions (3′UTRs) of target messenger RNAs (mRNAs), resulting in translational repression or mRNA degradation (26). Canonical miRNA biogenesis involves sequential processing by the Drosha and Dicer ribonucleases, followed by incorporation into the RNA-induced silencing complex (RISC) (27). However, a single miRNA can regulate multiple target genes simultaneously, enabling broad modulation of cellular pathways and signaling networks (28).

Long non-coding RNAs (lncRNAs) are transcripts longer than 200 nucleotides with little or no protein-coding potential. lncRNAs exhibit remarkable functional diversity and may regulate gene expression at the epigenetic, transcriptional, and post-transcriptional levels (29). They can function as molecular scaffolds, transcriptional regulators, chromatin modifiers, or competing endogenous RNAs (ceRNAs) that sponge miRNAs, thereby influencing downstream target expression (30). Compared with miRNAs, lncRNAs generally show more tissue- and cell-specific expression patterns, making them particularly attractive as disease biomarkers and therapeutic targets (31).

Circular RNAs (circRNAs) are another important class of ncRNAs generated by back-splicing events that covalently join the 3′ and 5′ ends of RNA molecules, producing a stable circular structure without free ends (32). This circular conformation renders circRNAs highly resistant to exonuclease-mediated degradation and contributes to their enhanced stability in tissues and biological fluids (33). Functionally, circRNAs commonly act as miRNA sponges, regulate transcription, interact with RNA-binding proteins, and, in some cases, undergo cap-independent translation (34). In addition to these major ncRNA classes, other non-coding transcripts, such as small nucleolar RNAs (snoRNAs), PIWI-interacting RNAs (piRNAs), and transfer RNA-derived fragments (tRFs), are emerging as important regulators of cellular physiology and disease pathogenesis, although their roles in skeletal disorders remain underexplored (35).

**Figure 1:**
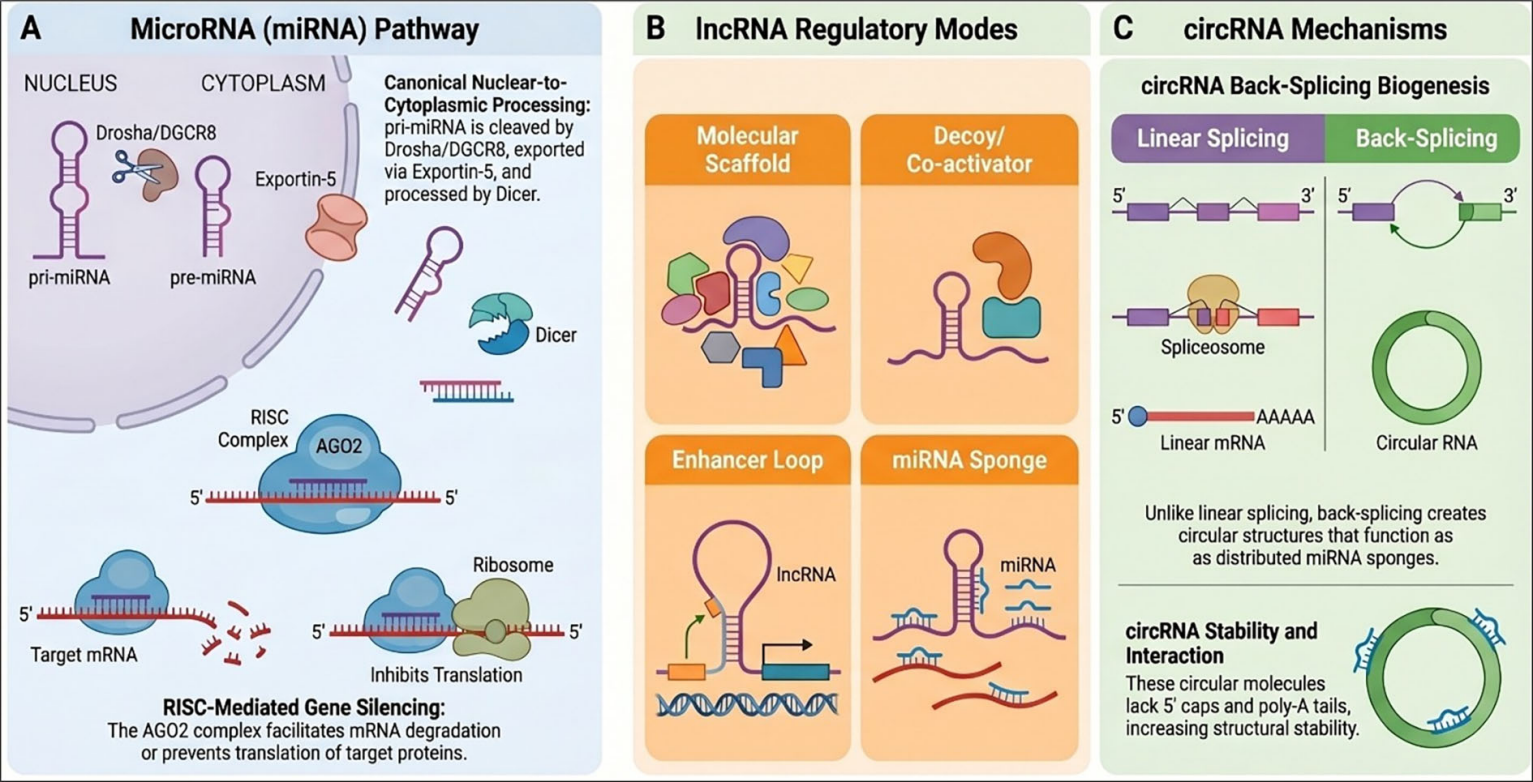
Biogenesis and regulatory mechanisms of major ncRNA classes. **(A)** illustrates canonical miRNA processing from pri-miRNA through Drosha/DGCR8 cleavage, nuclear export via Exportin-5, Dicer processing, and RISC-mediated mRNA degradation or translational repression. **(B)** depicts the four principal lncRNA regulatory modes: scaffold, decoy/co-activator, enhancer loop, and miRNA sponge. **(C)** contrasts linear splicing with back-splicing biogenesis of circRNAs and illustrates their function as distributed miRNA sponges.

The biogenesis pathways of ncRNAs illustrate distinct molecular routes that underlie their biogenesis and diverse regulatory functions. miRNAs undergo canonical nuclear-to-cytoplasmic processing, beginning as primary transcripts that are cleaved by Drosha/DGCR8, exported via Exportin-5, and further processed by Dicer before assembling into the RISC complex to silence target mRNAs (26, 27). lncRNAs are transcribed as stable transcripts and act through multiple modes, including serving as scaffolds for protein complexes, functioning as decoys or co-activators, forming enhancer loops, or sequestering miRNAs (29, 30). Circular RNAs (circRNAs) arise from back-splicing, forming covalently closed loops that lack 5′ caps and poly-A tails, conferring enhanced stability and enabling them to act as distributed miRNA sponges (32, 34). Collectively, these mechanisms underscore the broad regulatory potential of ncRNAs and their emerging significance in disease-associated gene regulatory networks, as summarized in **Figure 1**.

## 3 Role of non-coding RNA in bone physiology and health

In normal bone physiology, ncRNAs function as critical post-transcriptional and epigenetic regulators that maintain skeletal homeostasis by coordinating the tightly regulated balance between bone formation and bone resorption (9, 36). Bone remodeling is a dynamic and continuous process involving the coordinated activities of osteoblasts, osteoclasts, and osteocytes, and ncRNAs have emerged as key modulators of the signaling pathways governing these cellular interactions (37). Through fine-tuning gene expression networks, these ncRNAs regulate osteogenic differentiation, extracellular matrix mineralization, osteoclastogenesis, mechanotransduction, and intercellular communication within the bone microenvironment (36, 38). miRNAs play particularly important roles in osteoblast differentiation and function. Osteogenic miRNAs such as miR-29b, miR-218, and miR-26a promote osteoblast maturation and matrix mineralization by activating Wnt/β-catenin and BMP/SMAD signaling pathways, as well as enhancing the expression of osteogenic transcription factors including RUNX2 and Osterix (39, 40). Conversely, anti-osteogenic miRNAs such as miR-214, miR-204, and miR-133a negatively regulate osteoblast lineage commitment, thereby preventing excessive or aberrant mineralization (41). Within the osteoclast compartment, miRNAs also regulate bone resorption processes. miR-503 and miR-146a inhibit osteoclastogenesis by targeting key mediators such as RANK and TRAF6, whereas miR-21 and miR-148a promote osteoclast differentiation through amplification of RANKL/NFATc1 signaling pathways (42, 43). lncRNAs provide an additional level of regulatory complexity in bone biology. Several lncRNAs, including H19, MALAT1, and MEG3, positively regulate osteoblast differentiation through competing endogenous RNA (ceRNA) mechanisms that relieve miRNA-mediated suppression of osteogenic genes and Wnt signaling components (44, 45). In contrast, lncRNA NRON negatively regulates osteoclast formation by maintaining NFATc1 in an inactive cytoplasmic state through interactions with DYRK1A and RCAN proteins, thereby preventing excessive bone resorption (46). Circular RNAs (circRNAs) also contribute significantly to skeletal homeostasis, including ciRS-7 and circRNA_0076906, which promote osteogenic differentiation by sponging miR-7 and miR-182-5p, respectively, thereby sustaining the expression of pro-osteogenic transcription factors and signaling molecules (47). Beyond intracellular regulation, ncRNAs are increasingly recognized as important mediators of intercellular communication within the bone microenvironment. Extracellular vesicle-derived ncRNAs facilitate osteoblast–osteoclast coupling and coordinate bone remodeling activities. Osteoclast-derived miR-30a-3p and osteoblast-derived miR-503-3p have been shown to reciprocally regulate the differentiation and activity of remodeling partner cells, thereby maintaining skeletal equilibrium (48). Osteocytes, the principal mechanosensory cells of bone, also utilize ncRNAs such as miR-23a and lncRNA GAS5 to transduce mechanical stimuli, regulate mechanoadaptive responses, and protect against apoptosis by modulating PI3K/Akt signaling (49).

## 4 Role of ncRNAs in bone diseases

The skeletal system is affected by a wide spectrum of pathological disorders, in which dysregulation of ncRNAs has emerged as a critical molecular mechanism. Increasing evidence demonstrates that miRNAs, lncRNAs, and circRNAs regulate key cellular and signaling pathways involved in bone development, remodeling, inflammation, and degeneration (10, 50). In osteoporosis (OP), ncRNAs play pivotal roles in maintaining the balance between osteoblast-mediated bone formation and osteoclast-mediated bone resorption. Aberrant expression of ncRNAs contributes to reduced bone mineral density and increased fracture susceptibility by modulating pathways such as RUNX2, Wnt/β-catenin, RANK/RANKL/OPG, and NFATc1 signaling (51). Similarly, in rheumatoid arthritis (RA), ncRNAs regulate inflammatory cytokine production, immune-cell activation, synovial hyperplasia, and joint destruction, thereby influencing disease severity and progression (4). In osteosarcoma and other bone tumors, altered ncRNA expression has been associated with tumor proliferation, metastasis, angiogenesis, and chemoresistance, highlighting the importance of these ncRNAs in tumor biology and therapeutic targeting. Furthermore, ncRNAs have also been implicated in fracture healing, intervertebral disc degeneration, osteonecrosis, and other musculoskeletal disorders by regulating cell proliferation, apoptosis, extracellular matrix remodeling, and inflammatory signaling (10, 50). Among these skeletal disorders, OA is one of the most prevalent and debilitating degenerative joint diseases, in which ncRNAs orchestrate dysfunction in chondrocytes, synovial fibroblasts, and subchondral osteoblasts. Dysregulated ncRNA-mediated competing endogenous RNA (ceRNA) networks, along with aberrant modulation of signaling pathways such as Wnt/β-catenin, TGF-β/BMP, PI3K/Akt, and NF-κB, contribute significantly to cartilage degradation, synovial inflammation, and subchondral bone remodeling during OA progression (4, 11–13). The clinical relevance of ncRNAs in bone diseases is further strengthened by their stability in accessible biofluids and their emerging utility as diagnostic biomarkers and therapeutic targets (4, 50). The present review focuses on the roles of non-coding RNAs, viz., miRNAs, lncRNAs, and circRNAs, in OA, highlighting their mechanistic involvement in OA pathogenesis, their potential as circulating biomarkers, and the current advances and challenges associated with ncRNA-based therapeutic strategies for OA management.

## 5 Osteoarthritis pathogenesis

OA is a multifactorial and progressive degenerative joint disorder characterized by articular cartilage degradation, synovial inflammation, subchondral bone remodeling, osteophyte formation, and gradual loss of joint function (1, 2). Once considered a disease limited primarily to cartilage, OA is now recognized as a whole-joint pathology involving complex interactions among chondrocytes, synovial fibroblasts, immune cells, and subchondral bone cells (3). Multiple factors, including aging, mechanical stress, obesity, genetic predisposition, metabolic dysfunction, and chronic low-grade inflammation, collectively contribute to disease initiation and progression (1, 4).

At the molecular level, OA is characterized by disruption of the balance between anabolic and catabolic processes within the joint microenvironment. Excessive production of matrix-degrading enzymes, including matrix metalloproteinases (MMPs) and ADAMTS proteases, leads to degradation of extracellular matrix (ECM) components such as type II collagen and aggrecan (2, 52). Inflammatory mediators, including interleukin-1β (IL-1β), tumor necrosis factor-α (TNF-α), and reactive oxygen species, further amplify cartilage destruction, chondrocyte apoptosis, and inflammatory signaling by activating pathways such as NF-κB, MAPK, Wnt/β-catenin, and PI3K/Akt (4, 11–13).

Increasing evidence indicates that ncRNAs, including miRNAs, lncRNAs, and circRNAs, are key regulators of OA-associated molecular pathways and cellular responses. These ncRNAs influence extracellular matrix homeostasis, inflammatory signaling, apoptosis, autophagy, osteochondral remodeling, and intercellular communication, thereby contributing to disease progression and offering potential opportunities for biomarker discovery and targeted therapeutic development (11, 13).

### 5.1 Regulatory role of ncRNAs in OA

ncRNAs have emerged as major epigenetic regulators involved in nearly all stages of OA pathogenesis. By modulating gene expression at the transcriptional and post-transcriptional levels, miRNAs, lncRNAs, and circRNAs regulate critical biological processes underlying cartilage degeneration, synovial inflammation, and subchondral bone remodeling (53–56). **Table 1** summarizes the major OA-associated ncRNAs, including their expression patterns, molecular targets, signaling pathways, and functional relevance in OA progression.

**Table 1:** NcRNAs in osteoarthritis (OA): expression profile, regulatory mechanisms, and key findings across miRNAs, lncRNAs, and circRNAs.

ncRNA (type & name)	OA level/tissue	Mechanism	Study type	Key findings	References
MiRNAs					
miRNA miR-140-5p	Articular Cartilage ↓ (OA)	Suppresses ADAMTS5 (aggrecanase), reduces ECM degradation	Human tissue + Conditional KO mouse + *In vitro*	Most validated OA miRNA. KO mice show accelerated cartilage damage. Targeting ADAMTS5 directly limits aggrecan degradation.	(14–16)
miRNA miR-146a	Synovium/Chondrocytes ↑ (OA)	Inhibits IRAK1 & TRAF6 → suppresses NF-κB-driven inflammation	Human OA tissue + Murine *in vivo* + *In vitro*	Well-characterized anti-inflammatory axis. Consistently upregulated in OA synovium as a counter-regulatory response.	(17, 18, 57)
miRNA miR-21	Cartilage/Subchondral bone ↑ (OA)	Targets SMAD7 → amplifies TGF-β signaling; promotes catabolic activity; suppresses PDCD4 → osteoclastogenesis	*In vitro* (multiple lines) + Surgically-induced OA mouse model	Context-dependent: promotes cartilage degradation via TGF-β amplification; also linked to osteoclast activity. Replicated across multiple systems.	(19, 20)
miRNA miR-34a	Chondrocytes/Subchondral bone ↑ (OA)	Pro-apoptotic in chondrocytes; modulates osteoblast differentiation via DKK1 and Notch targets	*In vitro* + Animal models + Some human bone biopsy data	Drives chondrocyte apoptosis and alters bone remodeling. Multi-system evidence.	(58)
miRNA miR-9	Cartilage ↑ (OA)	Promotes catabolic gene expression in chondrocytes	*In vitro* + Human OA synovial fluid (expression)	Upregulated in OA; detected in synovial fluid. Less mechanistically detailed than miR-140 or miR-146a.	(19)
miRNA miR-155	Synovium ↑ (OA)	Promotes inflammatory gene expression in synovial tissue	Human OA synovial tissue (expression)	Paradoxically co-elevated with miR-146a in OA synovium; highlights complexity of inflammatory ncRNA networks.	(59)
miRNA miR-27a	Cartilage (OA)	Targets DKK1 (Wnt antagonist) → potentiates Wnt/β-catenin → chondrocyte hypertrophy	*In vitro* (cell-based)	Promotes chondrocyte hypertrophy and OA progression via Wnt activation.	(14)
miRNA miR-101/miR-373	Chondrocytes (OA)	Regulate MAPK/ERK inflammatory and catabolic signaling	*In vitro* (cell models)	Implicated in inflammatory signaling; evidence limited to cell models.	(20, 60)
miRNA – Biomarker miR-16, miR-21, miR-146a, miR-223	Blood/Plasma/Serum (OA patients)	Differential expression vs. healthy controls	Clinical (small cross-sectional; <100/group)	AUC ~0.7–0.9 reported. No prospective validation. No standardized protocol. Not yet clinically actionable.	(61, 62)
miRNA – Biomarker miR-140-5p, miR-9, miR-34a	Synovial Fluid (OA)	Differential expression vs. RA or healthy fluid	Clinical (small single-center)	Potentially more joint-specific than blood miRNAs; specificity unestablished without larger cohorts.	(19, 63, 64)
Long NcRNAs (lncRNAs)					
lncRNA HOTAIR	Cartilage/Synovium (OA)	Regulates histone methylation at cartilage gene loci (epigenetic); implicated in NF-κB-mediated gene regulation	*In vitro* (cell-based); limited human tissue data; biomarker (blood/SF)	Epigenetic reprogramming of OA cartilage genes. Most frequently reported OA lncRNA. Not validated in primary human OA tissue.	(21, 65, 66)
lncRNA MALAT1	Chondrocytes (OA)	Modulates chondrocyte autophagy and apoptosis pathways	*In vitro* (cell-based); biomarker (blood/SF)	Implicated in chondrocyte survival. Biomarker data preliminary; no independent replication.	(22, 67)
lncRNA NEAT1	Chondrocytes (OA)	ceRNA: sponges miR-141-3p → de-represses PTEN → modulates PI3K/AKT-mediated chondrocyte apoptosis	*In vitro* + Select animal models	ceRNA axis proposed. Evidence from immortalized lines; stoichiometric conditions *in vivo* unconfirmed. Not validated in primary human OA tissue.	(25)
lncRNA DANCR	Chondrocytes/Synovial macrophages (OA)	Modulates Wnt signaling in chondrocytes; synovial macrophage activation	*In vitro* (cell-based only)	Limited to cell models; no human OA tissue corroboration.	(68)
lncRNA THRIL	Synovial macrophages (OA)	Implicated in synovial macrophage activation and inflammation	*In vitro* (cell-based only)	Discovery-level evidence; no human OA synovial tissue corroboration.	(69)
lncRNA MEG3	Chondrocytes (OA)	Modulates Wnt/β-catenin activity in chondrocytes	*In vitro* (cell experiments)	Reported to modulate Wnt; evidence limited to cell experiments.	(70)
CircRNAs					
circRNA ciRS-7 (CDR1as)	Chondrocytes/OA tissue	ceRNA: sponges miR-7 (and OA-relevant miRNAs) → de-represses downstream targets	*In vitro* (cell models) + Transcriptomic profiling	Most cited OA circRNA. Mechanistic role in human OA tissue not established. Transcriptomic discovery stage.	(23, 24)
circRNA circRNA_0001946	Chondrocytes (OA)	Proposed ceRNA activity for OA-relevant miRNAs	*In vitro* (cell models) + Transcriptomic	Evidence from cell lines and profiling studies. *In vivo* relevance unsubstantiated.	(23)
circRNA – Biomarker Multiple (panels)	Blood/Synovial Fluid (OA)	Transcriptomic discovery: differential expression	Clinical (transcriptomic discovery; no targeted validation)	No individual circRNA biomarker has undergone rigorous diagnostic evaluation. Entirely hypothesis-generating.	(23)
miRNA – Therapeutic miR-140 mimic (atelocollagen)	Murine OA cartilage (intra-articular)	Restores miR-140-5p → suppresses ADAMTS5 → cartilage protection	Preclinical (rodent OA model – DMM/ACLT)	Proof-of-concept: prolonged cartilage protection in mice. Not tested in large animals or humans.	(16, 71, 72)
miRNA – Therapeutic MSC-derived exosomes (ncRNA cargo)	Rodent OA cartilage	Exosome-mediated delivery of protective ncRNAs to chondrocytes/synovium	Preclinical (rodent model)	Cartilage-protective effects in rodents. Large animal and human data absent.	(72, 73)
miRNA/lncRNA – Therapeutic AntagomiRs, ASOs, siRNAs	Chondrocytes/Synovial cells	Inhibit pathogenic ncRNAs or silence OA-driver genes	*In vitro* + Early preclinical	No ncRNA therapeutic in OA clinical trials. Delivery, off-target effects, and repeated dosing remain unsolved.	(74–76)

#### 5.1.1 ncRNAs in cartilage homeostasis and chondrocyte dysfunction

Maintenance of healthy articular cartilage depends on a tightly regulated balance between anabolic synthesis of ECM components, primarily type II collagen and aggrecan, and catabolic degradation mediated by MMPs and ADAMTS proteases (77, 78). In OA, disruption of this balance leads to progressive ECM degradation, chondrocyte apoptosis and hypertrophy, and impaired cartilage repair (79). ncRNAs are now recognized as central regulators of these pathological processes.

Among cartilage-protective miRNAs, miR-140-5p is among the most extensively characterized. Highly expressed in healthy chondrocytes, miR-140-5p directly suppresses ADAMTS5, a major aggrecanase that drives cartilage matrix degradation (14, 15). Reduced miR-140-5p expression is consistently observed in OA cartilage, and whole-body miR-140 knockout models show accelerated OA-like degeneration, underscoring its protective role *in vivo* (16). In contrast, miR-9, miR-34a, and miR-146a are frequently dysregulated in OA cartilage and contribute to inflammatory signaling, apoptosis, and catabolic gene expression linked to cartilage degeneration (17–19).

lncRNAs and circRNAs further contribute to chondrocyte dysfunction and ECM remodeling. lncRNA HOTAIR regulates histone methylation and inflammatory gene expression, whereas MALAT1 modulates chondrocyte autophagy, apoptosis, and survival pathways (21, 22). Similarly, circRNAs such as ciRS-7 and circRNA_0001946 participate in OA-associated signaling through competing endogenous RNA (ceRNA) mechanisms (23, 24). A major regulatory feature of OA-associated ncRNAs is the ceRNA network, in which lncRNAs and circRNAs act as molecular sponges that sequester specific miRNAs, thereby modulating downstream target gene expression (24). For example, lncRNA NEAT1 has been shown to sponge miR-141-3p, resulting in PTEN derepression and altered PI3K/Akt-mediated chondrocyte apoptosis in experimental OA models (25). Although many of these interactions have demonstrated functional significance *in vitro* and in preclinical models, further validation in human OA tissues and clinical studies remains necessary.

#### 5.1.2 ncRNAs in synovial inflammation

Synovial inflammation is increasingly recognized as a major contributor to OA-associated pain, inflammatory amplification, and structural disease progression (80). Activated synovial fibroblasts and infiltrating macrophages respond to damage-associated molecular patterns (DAMPs), IL-1β, and TNF-α by producing pro-inflammatory cytokines, chemokines, and matrix-degrading enzymes that exacerbate cartilage destruction (81). ncRNAs play critical roles in regulating these inflammatory cascades and associated signaling pathways, particularly NF-κB-mediated responses.

Among inflammatory miRNAs, miR-146a negatively regulates NF-κB signaling by targeting IRAK1 and TRAF6 (18, 57). Increased miR-146a expression in OA synovium is thought to reflect a compensatory anti-inflammatory response to chronic inflammation. Conversely, miR-155 is significantly upregulated in OA synovial tissues and promotes inflammatory gene expression and macrophage activation (59). These findings illustrate the complex, context-dependent roles of ncRNAs in OA-associated inflammation.

Several lncRNAs have also been implicated in synovial inflammation. lncRNAs such as THRIL and DANCR regulate inflammatory cytokine production and macrophage activation via NF-κB-associated pathways (68, 69). In addition, emerging circRNAs associated with synovitis have been identified through transcriptomic and *in vitro* studies, although their precise functional roles remain under investigation (23). Collectively, dysregulated ncRNA expression substantially contributes to persistent inflammatory signaling and the progression of synovial pathology in OA.

#### 5.1.3 ncRNAs in subchondral bone remodeling and osteochondral crosstalk

Subchondral bone remodeling is now recognized as an early, active component of OA pathogenesis rather than merely a secondary consequence of cartilage degeneration (82). Mechanical stress, inflammatory signaling, and osteochondral crosstalk modulate osteoblast and osteoclast activity, thereby increasing bone turnover, sclerosis, angiogenesis, and osteophyte formation within the osteochondral unit (83). ncRNAs involved in osteogenic and osteoclastogenic pathways, therefore, play important roles in OA-associated bone remodeling.

Several miRNAs implicated in skeletal remodeling also contribute to OA progression. miR-21 promotes osteoclastogenesis by suppressing PDCD4 and enhancing RANKL/NFATc1 signaling (20), whereas miR-34a regulates osteoblast differentiation and subchondral remodeling via DKK1- and Notch-associated pathways (58). Growing evidence suggests that lncRNAs and circRNAs regulate osteoblast differentiation, osteoclast activity, angiogenesis, and osteochondral communication through pathways including Wnt/β-catenin, PI3K/Akt, and TGF-β/BMP signaling (44, 45, 47, 84). Together, these findings demonstrate that ncRNAs coordinate pathological changes across cartilage, synovium, and subchondral bone, thereby contributing to the integrated progression of OA as a whole-joint disease.

## 6 Key signaling pathways regulated by ncRNAs in OA

**Figure 2:**
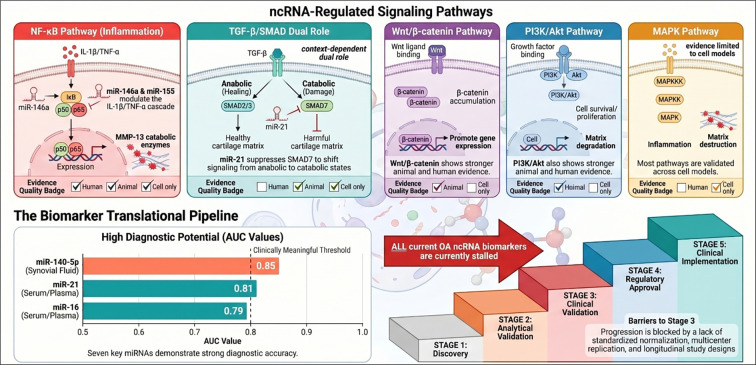
ncRNA-regulated signaling pathways and biomarker translation in OA. Five major OA-associated pathways (NF-κB, TGF-β/SMAD, Wnt/β-catenin, PI3K/Akt, and MAPK) are shown, along with their ncRNA regulators and the quality of evidence across experimental systems. The biomarker pipeline indicates that all current OA ncRNA candidates remain stalled at the analytical validation stage, with none achieving clinical implementation.

**Figure 3:**
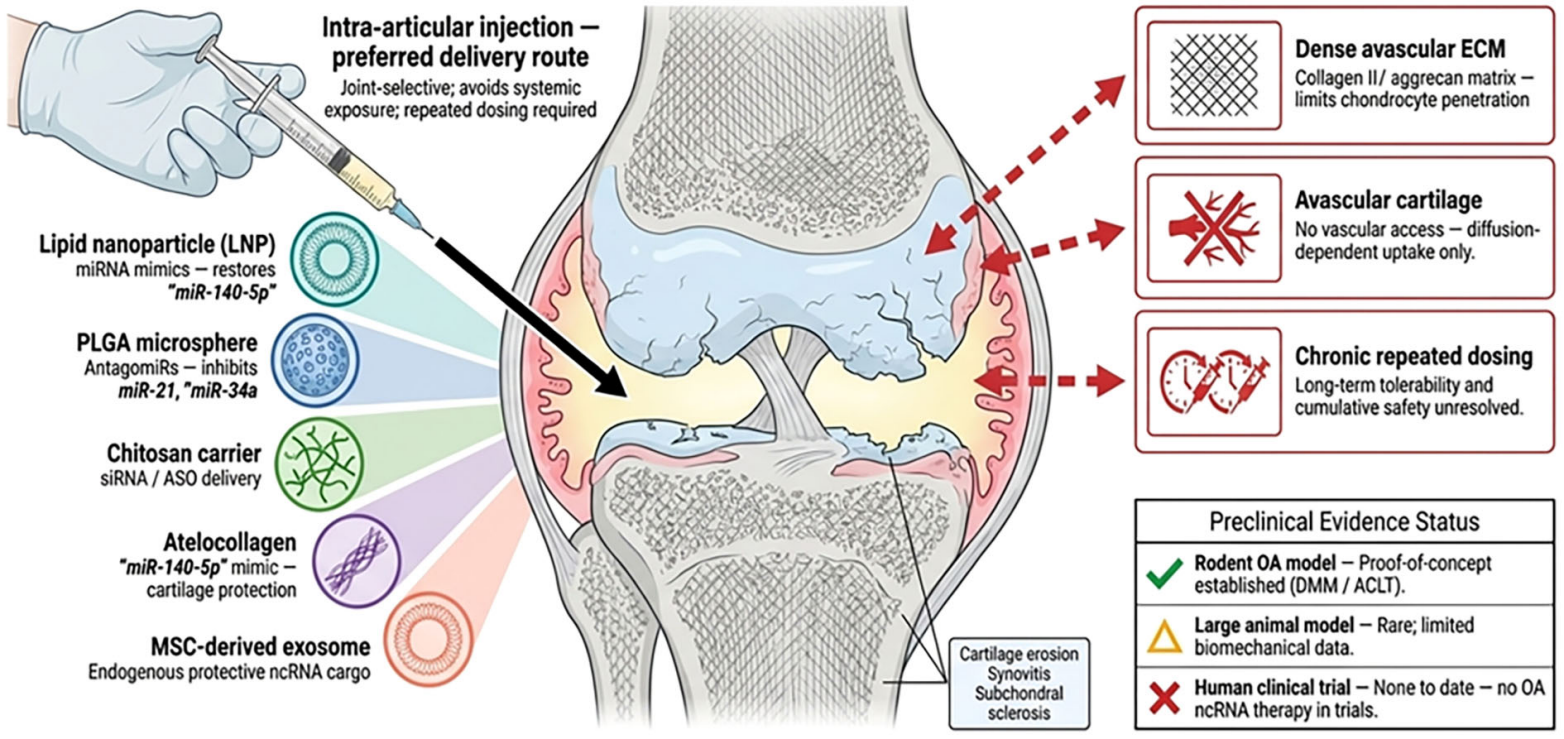
Intra-articular ncRNA therapeutic delivery strategies for OA. Five delivery vehicles (lipid nanoparticles, PLGA microspheres, chitosan carriers, atelocollagen complexes, and MSC-derived exosomes) are shown, along with their respective ncRNA cargo types and mechanisms. Key biological barriers, including the avascular cartilage matrix, limited vascular access, and the need for repeated dosing, are highlighted. All current evidence comes from preclinical rodent models; no OA ncRNA therapy has entered clinical trials.

The pathological progression of OA is driven by complex interactions among inflammatory, catabolic, apoptotic, and remodeling pathways, many of which are tightly regulated by ncRNAs. Increasing evidence shows that miRNAs, long ncRNAs (lncRNAs), and circRNAs modulate multiple signaling cascades involved in cartilage degeneration, synovial inflammation, and osteochondral remodeling. Among the most extensively studied pathways in OA are NF-κB, TGF-β/SMAD, Wnt/β-catenin, PI3K/Akt, and MAPK/ERK signaling pathways, each of which contributes to distinct yet interconnected aspects of OA pathogenesis (4, 84–89). A schematic overview of these ncRNA-regulated signaling pathways, their downstream pathological consequences, the quality of evidence across experimental systems, and the status of ncRNA biomarker clinical translation is presented in **Figure 2**. The figure further illustrates that, while ncRNAs regulate NF-κB and Wnt/β-catenin pathways, as supported by human, animal, and cell-based evidence, involvement of the MAPK pathway remains largely confined to cell models, underscoring the uneven depth of validation across pathways. Additionally, **Figure 3** highlights that all currently proposed OA ncRNA biomarkers remain stalled at the analytical validation stage, with none having achieved clinical implementation yet.

### 6.1 NF-κB signaling pathway

The nuclear factor-kappa B (NF-κB) pathway is one of the best-characterized inflammatory signaling cascades in OA and serves as a central regulator of cytokine-driven cartilage degeneration and synovial inflammation (90). Pro-inflammatory mediators such as interleukin-1β (IL-1β) and tumor necrosis factor-α (TNF-α) activate NF-κB signaling, promoting the transcription of matrix-degrading enzymes, including MMP-3, MMP-13, ADAMTS5, and cyclooxygenase-2 (COX-2), thereby accelerating extracellular matrix degradation and chondrocyte apoptosis (80, 91). Several ncRNAs regulate this pathway at multiple levels. Among the best-characterized anti-inflammatory miRNAs, miR-146a negatively regulates NF-κB signaling by directly targeting interleukin-1 receptor-associated kinase 1 (IRAK1) and TNF receptor-associated factor 6 (TRAF6), both critical upstream mediators of NF-κB activation (17, 18). Increased expression of miR-146a has been consistently reported in OA cartilage and synovial tissues, where it likely functions as a compensatory mechanism to limit excessive inflammation (17). Functional studies in human OA chondrocytes and murine OA models have confirmed that miR-146a suppresses inflammatory cytokine production and reduces cartilage degradation (18). Conversely, miR-155 acts as a pro-inflammatory regulator, enhancing NF-κB-mediated inflammatory responses and synovial macrophage activation (59). lncRNAs have also been implicated in NF-κB regulation. lncRNA HOTAIR has been reported to influence inflammatory gene expression by epigenetically modulating chromatin structure and NF-κB-associated transcriptional activity in chondrocytes and synovial cells (21, 65). Similarly, several circRNAs have been proposed to modulate NF-κB signaling through ceRNA-mediated sequestration of inflammatory miRNAs, although most evidence currently comes from *in vitro* studies and transcriptomic analyses (23).

### 6.2 TGF-β/SMAD signaling pathway

Transforming growth factor-beta (TGF-β)/SMAD signaling plays a dual, context-dependent role in cartilage biology and OA progression (87). Under physiological conditions, TGF-β signaling maintains cartilage homeostasis by promoting extracellular matrix synthesis, chondrocyte proliferation, and anabolic activity. However, aberrant or sustained TGF-β signaling in OA drives chondrocyte hypertrophy, osteophyte formation, fibrosis, and increased catabolic activity (88). Several ncRNAs regulate TGF-β/SMAD pathway activity in OA. miR-21 is among the most extensively studied OA-associated miRNAs linked to this pathway. By targeting SMAD7, an inhibitory SMAD protein that negatively regulates TGF-β signaling, miR-21 amplifies TGF-β pathway activation and contributes to cartilage matrix degradation and osteoclastogenesis (19, 20). Elevated miR-21 expression has been observed in OA cartilage and subchondral bone tissues, and its pathogenic role has been demonstrated in multiple cell-based studies and surgically induced OA mouse models (19, 20). In addition, lncRNAs and circRNAs that regulate SMAD signaling components via ceRNA mechanisms have been identified, although many remain insufficiently validated in primary human OA tissues.

### 6.3 Wnt/β-catenin signaling pathway

The Wnt/β-catenin signaling pathway is a major regulator of cartilage development, chondrocyte differentiation, and osteochondral homeostasis (84). Physiological Wnt activity is required for cartilage maintenance; however, aberrant activation of Wnt/β-catenin signaling in OA promotes chondrocyte hypertrophy, extracellular matrix degradation, osteophyte formation, and subchondral bone sclerosis (4). Multiple ncRNAs modulate this pathway during OA progression. miR-27a enhances Wnt/β-catenin signaling by directly targeting Dickkopf-related protein 1 (DKK1), a key Wnt antagonist (14). Upregulation of miR-27a has been linked to increased chondrocyte hypertrophy and catabolic activity in OA models. Similarly, lncRNAs such as DANCR and MEG3 have been reported to regulate Wnt signaling in chondrocytes and mesenchymal stem cells, primarily through ceRNA-mediated interactions with Wnt-associated miRNAs (68, 70). Several circRNAs have also been implicated in modulating β-catenin stability and downstream transcriptional activity, although mechanistic validation remains limited.

### 6.4 PI3K/Akt signaling pathway

The phosphatidylinositol 3-kinase (PI3K)/Akt signaling pathway is critical for regulating chondrocyte survival, proliferation, autophagy, and apoptosis (11). Dysregulation of PI3K/Akt signaling contributes to oxidative stress-induced apoptosis, inflammatory injury, and impaired cartilage repair during OA progression. Numerous ncRNA-mediated ceRNA networks targeting phosphatase and tensin homolog (PTEN), a negative regulator of PI3K/Akt signaling, have been identified in OA models (25). One well-characterized example is lncRNA NEAT1, which acts as a ceRNA for miR-141-3p, thereby relieving suppression of PTEN and modulating PI3K/Akt-mediated chondrocyte apoptosis (25). Similar ceRNA axes involving circRNAs and miRNAs that regulate PTEN/Akt signaling have also been reported in experimental OA systems. However, many of these findings are based on bioinformatic predictions, immortalized cell lines, or limited animal studies and require validation in primary human OA tissues and clinically relevant models.

### 6.5 MAPK/ERK signaling pathway

Mitogen-activated protein kinase (MAPK)/extracellular signal-regulated kinase (ERK) signaling is another key pathway involved in OA-associated inflammation and catabolic gene expression (13). IL-1β and mechanical stress activate MAPK/ERK signaling, inducing expression of MMPs, inflammatory cytokines, and apoptosis-related genes in chondrocytes and synovial cells (89). Several miRNAs, including miR-101 and miR-373, have been implicated in regulating MAPK/ERK-dependent inflammatory signaling and cartilage catabolism in OA cell models (20, 60). Although numerous ncRNA-mediated signaling mechanisms have been proposed in OA, many mechanistic claims currently derive from bioinformatic analyses, single-cell-line experiments, or limited preclinical studies. Therefore, further validation in primary human tissues, large-animal models, and longitudinal clinical studies is essential before these ncRNA-regulated pathways can be translated into reliable biomarkers or therapeutic targets for OA management.

## 7 Diagnostic potential: ncRNAs as biomarkers in OA

The remarkable stability, tissue specificity, and detectability of ncRNAs in accessible biofluids such as blood, serum, plasma, synovial fluid, and urine have generated considerable interest in their potential as minimally invasive biomarkers for OA diagnosis, disease stratification, prognosis, and therapeutic monitoring (3, 5). Unlike conventional radiographic assessment, which primarily detects structural changes at relatively advanced disease stages, ncRNA-based biomarkers may provide insights into early molecular alterations linked to cartilage degradation, synovial inflammation, and osteochondral remodeling (1). Consequently, extensive efforts have focused on identifying disease-associated miRNAs, lncRNAs, and circRNAs that could serve as clinically useful biomarkers for OA.

**Table 2** offers a comprehensive overview of OA-associated ncRNA biomarkers under investigation, summarizing major circulating and tissue-derived ncRNAs, their biofluid sources, expression patterns, molecular functions, study types, and reported diagnostic significance. The table also highlights key methodological considerations, including sample type, cohort size, validation level, and translational limitations for each proposed biomarker. Additionally, it categorizes ncRNAs by their roles as pathogenic regulators, diagnostic candidates, and emerging therapeutic targets, thereby providing an integrated overview of the current state of ncRNA biomarker research in OA.

**Table 2:** Diagnostic potential of circulating ncRNAs in Osteoarthritis: expression profiles, study designs, and limitations.

ncRNA	Biofluid	OA expression	N (OA/Ctrl)	AUC	Study design	Limitation	Ref
MiRNAs							
miR-21	Serum	↑ OA vs HC	85/80	0.81	Cross-sectional; single-center	No validation cohort; no standard normalization	(61)
miR-146a	Serum/Plasma	↑ OA vs HC	62/58	0.77	Cross-sectional; single-center	Heterogeneous controls; no replication	(62)
miR-16	Serum	↑ OA vs HC	90/85	0.79	Cross-sectional	Small cohort; no independent validation	(61)
miR-223	Plasma	↑ OA vs HC	74/70	0.73	Cross-sectional; single-center	No prospective design; limited specificity data	(62)
miR-140-5p	Serum	↓ OA vs HC	45/40	0.85	Cross-sectional; OA vs RA comparison	Very small cohort; single center; no replication	(63)
miR-9	Synovial fluid	↑ OA vs HC	38/35	NR	Expression profiling only	No AUC reported; discovery-level only	(19)
miR-34a	Synovial fluid	↑ OA vs RA	50/45	0.76	Cross-sectional	OA vs RA comparison; no healthy controls	(64)
Long NcRNAs (lncRNAs)							
HOTAIR	Blood/PBMCs	↑ OA vs HC	40/38	NR	Transcriptomic profiling; small cohort	No AUC; no replication; preliminary only	(66)
MALAT1	Serum	↑ OA vs HC	35/32	NR	Expression profiling only	Discovery-stage; no targeted validation	(67)
NEAT1	Serum	↑ OA vs HC	42/40	NR	Cell-based + small clinical sample	No prospective design; no validation cohort	(25)
CircRNAs							
ciRS-7 (CDR1as)	Synovial fluid	Dysregulated	NR	NR	Transcriptomic discovery only	No diagnostic evaluation; hypothesis-generating only	(23)
circRNA panels (multiple)	Blood/SF	Variable	NR	NR	Transcriptomic profiling	No individual circRNA validated; no AUC reported	(92)

Circulating ncRNA biomarker studies in OA. Summary of reported miRNAs, lncRNAs, and circRNAs detected in patient biofluids, showing their differential expression in OA versus controls, diagnostic performance, study designs, and key limitations. NR, not reported; HC, healthy controls; SF, synovial fluid; PBMCs, peripheral blood mononuclear cells; AUC, area under the receiver operating characteristic curve. ↑, increased expression in OA; ↓, decreased expression in OA, relative to the comparison group shown.

Among circulating miRNAs, miR-16, miR-21, miR-146a, and miR-223 are among the most frequently reported as differentially expressed in serum or plasma samples from OA patients compared with healthy controls (61, 86). Several studies have reported receiver operating characteristic (ROC) curve area under the curve (AUC) values ranging from approximately 0.70 to 0.90, suggesting potential diagnostic utility (62). However, these findings should be interpreted cautiously, given substantial methodological limitations across studies. Most published biomarker investigations have involved relatively small cohorts, often fewer than 100 participants per group, and have employed cross-sectional rather than longitudinal study designs (77). Furthermore, considerable heterogeneity exists in patient selection criteria, disease staging, RNA isolation methods, normalization strategies, and reference gene selection, thereby limiting reproducibility and inter-study comparability (56). As highlighted in **Table 2**, very few ncRNA biomarkers have undergone independent external validation or prospective clinical evaluation, emphasizing that currently reported diagnostic performance metrics remain preliminary rather than clinically actionable.

Synovial fluid-derived ncRNAs have also attracted attention as potentially more joint-specific biomarkers due to their direct proximity to affected tissues and pathological processes in the OA joint microenvironment (78). Several miRNAs, including miR-140-5p, miR-9, and miR-34a, have been detected in OA synovial fluid and show differential expression compared with healthy controls or with inflammatory arthropathies such as rheumatoid arthritis (RA) (63, 64). miR-140-5p has attracted significant interest for its cartilage-protective role and for its reduced expression in OA cartilage and synovial fluid (14, 58). Nevertheless, most synovial fluid biomarker studies remain limited by small sample sizes, single-center recruitment, lack of standardized sample-processing protocols, and the absence of longitudinal replication cohorts (78). Consequently, the specificity, sensitivity, and prognostic value of these candidate biomarkers remain insufficiently established.

Long ncRNAs such as HOTAIR, MALAT1, and NEAT1 have likewise been reported to be differentially expressed in blood and synovial fluid samples from patients with OA (66, 67). These lncRNAs are implicated in inflammatory signaling, chondrocyte apoptosis, and extracellular matrix remodeling, suggesting potential utility as both mechanistic and diagnostic biomarkers (53). However, the current evidence base for lncRNA biomarkers remains highly preliminary, with most findings from isolated studies with limited patient numbers and lacking independent replication. Similarly, circRNA biomarker research in OA remains in its exploratory stage. Most currently identified circRNA candidates have emerged from transcriptomic profiling and high-throughput sequencing studies rather than from targeted validation analyses (23). Although several circRNAs exhibit disease-specific expression patterns and favorable molecular stability, no circRNA biomarker has yet undergone rigorous multicenter diagnostic evaluation or clinical validation.

In addition to diagnostic applications, ncRNAs may also have prognostic and therapeutic monitoring value in OA. Because ncRNA expression profiles are dynamically regulated during inflammation, cartilage degeneration, and tissue remodeling, longitudinal changes in circulating ncRNAs may reflect disease progression or responses to therapeutic interventions (3). However, these applications remain largely hypothetical because prospective longitudinal studies and standardized analytical methodologies are currently lacking.

Addressing these limitations will require implementing robust, standardized pre-analytical and analytical protocols, including harmonized sample collection procedures, optimized RNA extraction methods, validated normalization controls, and reproducible quantitative platforms (1, 56). Importantly, future biomarker studies must include adequately powered multicenter cohorts, standardized clinical phenotyping, and longitudinal validation designs with clearly defined clinical endpoints. Until this methodological rigor is achieved, reported ncRNA biomarker findings should be regarded as hypothesis-generating rather than as clinically validated tools for OA diagnosis or management. At present, no ncRNA biomarker has been incorporated into routine clinical practice for OA.

## 8 Therapeutic potential of ncRNA-based strategies in OA

The growing recognition of ncRNAs as central regulators of OA pathogenesis has spurred substantial interest in developing RNA-based therapeutic strategies to modulate disease-associated molecular pathways (15). Because ncRNAs regulate multiple interconnected signaling cascades involved in cartilage degradation, inflammation, apoptosis, autophagy, and osteochondral remodeling, targeting these pathways could simultaneously modulate multiple pathogenic mechanisms rather than targeting individual downstream effectors (16). Consequently, multiple ncRNA-based therapeutic approaches have been explored in preclinical OA models, including miRNA mimics to restore downregulated protective miRNAs, antagomiRs or antimiRs to inhibit pathogenic miRNAs, antisense oligonucleotides (ASOs) targeting lncRNAs, and small interfering RNAs (siRNAs) to silence disease-associated transcripts (74, 75).

**Figure 3** summarizes an overview of currently investigated ncRNA-based therapeutic strategies for OA, illustrating the major therapeutic ncRNAs, their molecular targets, delivery systems, mechanisms of action, and biological effects within the osteoarthritic joint. It highlights various delivery platforms under investigation, including lipid nanoparticles, polymeric microspheres, hydrogel-based carriers, and extracellular vesicle or exosome-mediated systems. In addition, it summarizes the principal pathological processes targeted by ncRNA therapeutics, including cartilage degradation, synovial inflammation, chondrocyte apoptosis, osteoclast activation, and osteochondral remodeling. It also outlines the current experimental status of these approaches, emphasizing that most available evidence is derived from *in vitro* studies and preclinical animal models rather than human clinical trials.

### 8.1 miRNA-based therapeutic strategies for OA

Among ncRNA therapeutics, miRNA-based approaches are the most extensively investigated in OA. Protective miRNAs that are downregulated during OA progression, such as miR-140-5p, miR-26a, and miR-146a, are attractive candidates for replacement therapy using synthetic miRNA mimics (14). miR-140-5p has emerged as a particularly promising therapeutic candidate because of its critical role in maintaining cartilage homeostasis by suppressing ADAMTS5 and matrix-degrading pathways (14, 15). Intra-articular administration of miR-140 mimics encapsulated in atelocollagen has been shown to reduce cartilage degeneration and preserve joint structure in murine OA models, providing important proof of concept for miRNA replacement therapy (71). Similarly, miR-146a mimics have demonstrated anti-inflammatory effects by inhibiting NF-κB signaling in experimental OA models (18).

Conversely, inhibiting pathogenic miRNAs with antagomiRs or antimiRs is another major therapeutic strategy. miRNAs such as miR-21, miR-34a, and miR-155 are frequently upregulated in OA and contribute to inflammatory signaling, osteoclastogenesis, chondrocyte apoptosis, and extracellular matrix degradation (19, 59). Experimental inhibition of these pathogenic miRNAs has been associated with reduced inflammatory cytokine production, reduced cartilage destruction, and improved chondrocyte survival in preclinical studies (17). However, because individual miRNAs often regulate hundreds of target genes across multiple tissues, concerns remain about potential off-target effects and unintended systemic consequences of therapeutic modulation.

### 8.2 lncRNA and siRNA-based therapeutics

Long non-coding RNAs (lncRNAs) have also emerged as potential therapeutic targets in OA due to their roles in chromatin remodeling, transcriptional regulation, and competing endogenous RNA (ceRNA) networks (18). Antisense oligonucleotides designed to target pathogenic lncRNAs, such as HOTAIR and NEAT1, have been explored as candidate strategies in cell-based OA models, with preliminary evidence suggesting anti-inflammatory effects (21, 25). Similarly, siRNA-mediated suppression of catabolic mediators, inflammatory cytokines, and signaling molecules involved in OA progression has been shown to reduce cartilage degradation and synovial inflammation in preclinical models (19). Nevertheless, efficient intracellular delivery and sustained transcript silencing remain significant challenges for clinical translation.

### 8.3 Delivery systems for ncRNA therapeutics

The primary obstacle to the clinical application of ncRNA therapeutics in OA is the development of safe, stable, and joint-selective delivery systems that protect RNA molecules from rapid degradation and ensure efficient uptake by target cells (74, 75). Because OA primarily affects a localized joint environment, intra-articular delivery is generally considered the preferred route of administration, enabling high local drug concentrations while minimizing systemic exposure and off-target toxicity (71). A wide range of nanoparticle-based delivery systems has therefore been explored for OA-directed ncRNA delivery. Lipid nanoparticles (LNPs), which gained substantial attention following their successful application in mRNA vaccine platforms, have been investigated for intra-articular delivery of miRNA mimics and siRNAs to chondrocytes and synovial cells (74). Poly(lactic-co-glycolic acid) (PLGA) microspheres and nanoparticles provide controlled release and prolonged intra-articular retention, thereby improving therapeutic stability and sustained RNA delivery (75). Chitosan-based carriers have similarly demonstrated favorable biocompatibility and cartilage-targeting potential in experimental OA systems (76). Hydrogel-based scaffolds and injectable biomaterials are also being investigated as sustained-release reservoirs for ncRNA therapeutics within the joint microenvironment (72). These delivery systems and their mechanisms of action are further illustrated in **Figure 3**, which compares their structural features, release properties, and therapeutic applications in OA models. Extracellular vesicle- and exosome-mediated delivery have emerged as particularly attractive strategies for ncRNA therapeutics in OA. Mesenchymal stem cell (MSC)-derived exosomes naturally contain bioactive miRNAs and possess intrinsic tissue-protective and immunomodulatory properties (80). Several preclinical studies have demonstrated that MSC-derived exosomes enriched with cartilage-protective miRNAs can reduce inflammation, inhibit chondrocyte apoptosis, and promote cartilage repair in rodent OA models (72, 73). Compared with synthetic nanoparticle systems, exosomes may offer advantages such as improved biocompatibility, lower immunogenicity, enhanced cellular uptake, and natural tissue-targeting capabilities (81). However, significant challenges remain in standardizing exosome isolation, scaling up manufacturing, ensuring cargo consistency, and navigating regulatory approval pathways.

## 9 Limitations in preclinical studies and challenges in clinical translatability

Despite encouraging experimental findings, the current evidence supporting ncRNA therapeutics for OA remains largely preclinical and should be interpreted cautiously. Most available studies have been conducted in cell culture systems or rodent OA models, particularly the destabilization of the medial meniscus (DMM) and anterior cruciate ligament transection (ACLT) models (57, 82). Although these systems provide valuable mechanistic and proof-of-concept insights, they do not fully replicate the complexity of human OA pathophysiology. Rodent joints differ substantially from human joints in anatomy, biomechanics, cartilage thickness, immune responses, and healing capacity (59). Consequently, therapeutic efficacy observed in murine models may not translate directly to human disease.

Large-animal models, including sheep, goats, horses, and canine OA systems, may provide more clinically relevant biomechanical and anatomical environments for evaluating intra-articular ncRNA delivery and therapeutic durability (82, 83). However, such studies remain relatively uncommon because of high costs, technical complexity, and ethical considerations. Importantly, no ncRNA-based therapeutic has yet advanced to clinical trials specifically for OA treatment. Therefore, currently reported therapeutic successes should be regarded as preliminary proof-of-concept findings rather than evidence of clinical readiness. Substantial additional work, including standardized delivery systems, long-term safety evaluation, pharmacokinetic characterization, large-animal validation, and ultimately well-designed human clinical trials, will be required before ncRNA-based therapeutics can become viable treatment options for OA.

Despite growing evidence supporting the role of ncRNAs in OA, their clinical translation remains challenging. Although numerous miRNAs, long ncRNAs (lncRNAs), and circRNAs have been identified as potential biomarkers and therapeutic targets, only a few have advanced beyond preclinical stages (50, 69). Major barriers include a lack of biomarker standardization, inefficient delivery systems, safety concerns, regulatory uncertainties, and limited long-term clinical validation.

A major limitation in ncRNA biomarker development is the lack of standardized protocols for sample collection, RNA isolation, normalization, and analysis (56). Variability in pre-analytical and analytical procedures often leads to inconsistent results across studies (83). Circulating ncRNA expression is also influenced by factors such as age, obesity, inflammation, medications, and comorbidities, thereby complicating the identification of OA-specific signatures (53, 93, 94). OA heterogeneity further complicates biomarker validation, and most studies are limited by small cohorts and insufficient longitudinal validation.

For ncRNA therapeutics, efficient, tissue-specific delivery remains a critical challenge. The dense, avascular cartilage matrix limits therapeutic molecules’ penetration into chondrocytes (74, 75). Chemical modifications, such as phosphorothioate backbones and locked nucleic acid (LNA) chemistry, improve RNA stability and target affinity but may also increase toxicity, immunogenicity, and manufacturing complexity (74, 75). Because OA is a chronic disease, repeated intra-articular administration may be required, raising concerns about long-term safety, cumulative toxicity, and patient compliance (71, 74). Furthermore, ncRNAs regulate multiple signaling pathways simultaneously, increasing the risk of unintended off-target effects (26).

Regulatory and manufacturing challenges also impede clinical implementation. Although RNA therapeutics have shown success in other diseases, regulatory frameworks for intra-articular ncRNA therapies for OA remain underdeveloped (74, 76). Large-scale manufacturing of ncRNA molecules and delivery systems requires stringent quality control, reproducibility, and stability. Exosome-based therapies also raise concerns about donor variability and cargo heterogeneity (72, 73).

Future progress will depend on interdisciplinary collaboration, standardized methodologies, advanced delivery platforms, and large multicenter clinical studies. Emerging technologies, including artificial intelligence, spatial transcriptomics, and multi-omics integration, may enhance biomarker precision and therapeutic targeting in OA (50, 69). Although several translational barriers remain, continued advances in RNA therapeutics and nanotechnology offer promising opportunities for ncRNA-based precision medicine approaches in OA.

## 10 Discussion

The present review highlights that ncRNAs are important regulators of OA pathogenesis, influencing cartilage degradation, synovial inflammation, extracellular matrix remodeling, apoptosis, and subchondral bone changes through multiple interconnected signaling pathways (86, 95). Among the three major ncRNA classes, miRNAs currently have the strongest mechanistic and translational evidence, whereas long ncRNAs (lncRNAs) and circular RNAs (circRNAs) remain largely in the exploratory and discovery stages (13, 53).

Among miRNAs, miR-140-5p emerges as the most biologically validated candidate, with consistent evidence from human OA cartilage, whole-body knockout mouse models, and functional cell studies demonstrating its regulation of ADAMTS5-mediated cartilage degradation (14–16). Similarly, miR-146a has shown reproducible anti-inflammatory effects by modulating the IRAK1/TRAF6/NF-κB signaling axis in chondrocytes and experimental OA models (17, 18). Other miRNAs, including miR-21 and miR-34a, have also been associated with chondrocyte apoptosis, catabolic signaling, and inflammatory responses, although some findings remain context-dependent (64, 70). In contrast, lncRNAs such as HOTAIR, MALAT1, and NEAT1, as well as circRNAs including ciRS-7, are supported largely by transcriptomic profiling and cell-based studies, with limited validation in primary human OA tissues or *in vivo* systems (21–23).

This review also highlights that ncRNAs regulate several major OA-associated signaling pathways, including NF-κB, TGF-β/SMAD, PI3K/Akt, MAPK, and Wnt/β-catenin (4, 11, 13, 84, 86–89). Among these, the miR-146a/NF-κB and miR-140/ADAMTS5 axes appear to be the most consistently validated mechanisms across experimental platforms (20, 80). The competing endogenous RNA (ceRNA) hypothesis, involving lncRNA- and circRNA-mediated miRNA sponging, has attracted significant attention; however, much of the evidence remains indirect and warrants cautious interpretation, as mechanistic validation is still insufficient (25, 96).

The biomarker potential of circulating ncRNAs is another promising aspect discussed in this review. Several miRNAs, including miR-21, miR-16, miR-146a, miR-140-5p, and miR-223, show altered expression in the serum, plasma, or synovial fluid of OA patients (62, 63). Nevertheless, most biomarker studies remain limited by small sample sizes, cross-sectional designs, a lack of standardized normalization methods, and insufficient external validation (56, 77). Biological variability related to age, obesity, metabolic status, inflammation, and OA heterogeneity further complicates the development of clinically reliable ncRNA signatures (56, 77, 93, 94). Consequently, no ncRNA biomarker has yet achieved routine clinical applicability in OA.

From a therapeutic perspective, ncRNA-based strategies offer the potential to target disease-driving molecular pathways rather than merely controlling symptoms (5, 69). Preclinical studies using miRNA mimics, antisense oligonucleotides, siRNAs, nanoparticles, and exosome-based delivery systems have shown promising results in reducing inflammation and cartilage degeneration (69, 97). However, translation into clinical practice remains limited by major challenges, including poor cartilage penetration, RNA instability, off-target effects, immunogenicity, the need for repeated dosing, and long-term safety concerns (60, 61, 82). In addition, most therapeutic evidence comes from rodent OA models, which do not fully replicate the anatomical and biomechanical complexity of human OA (98).

Overall, the evidence reviewed here suggests that ncRNAs have considerable potential as biomarkers and therapeutic targets in OA, but the field remains largely preclinical. Future progress will require standardized biomarker methodologies, robust multicenter validation studies, improved cartilage-targeted delivery systems, large-animal translational models, and greater mechanistic clarity regarding ncRNA regulatory networks (56, 65, 69). Emerging technologies such as single-cell and spatial transcriptomics, artificial intelligence-driven biomarker analysis, and multi-omics integration may further enhance the precision and clinical applicability of ncRNA-based approaches in OA (65, 66).

## 11 Conclusion and future directions

Non-coding RNAs (ncRNAs) have emerged as key regulators of OA pathogenesis by modulating major OA-associated signaling pathways. Experimental studies have highlighted the roles of miRNAs such as miR-140-5p, miR-146a, miR-21, and miR-34a in regulating cartilage degradation, inflammatory signaling, osteoclastogenesis, and chondrocyte survival in OA. Similarly, lncRNAs and circRNAs, including HOTAIR, MALAT1, NEAT1, and ciRS-7, have been implicated in OA progression, although many findings still require validation in human OA tissues. Circulating ncRNAs in serum, plasma, and synovial fluid also show promise as minimally invasive biomarkers for OA diagnosis and monitoring. In addition, ncRNA-based therapeutics, including miRNA mimics, antagomiRs, antisense oligonucleotides, siRNA platforms, nanoparticle carriers, and exosome-mediated delivery systems, have demonstrated encouraging cartilage-protective and anti-inflammatory effects in preclinical OA models. However, challenges related to delivery efficiency, tissue specificity, methodological heterogeneity, small cohort sizes, and long-term safety continue to limit clinical translation.

Future studies should prioritize large, prospectively designed biomarker studies with standardized analytical pipelines and independent validation cohorts to improve reproducibility and translational reliability. Integrating ncRNA profiling with multi-omics approaches, including proteomics, metabolomics, genomics, and imaging biomarkers, may help develop more robust diagnostic signatures. Mechanistic investigations should increasingly use primary human chondrocytes, patient-derived OA tissues, and three-dimensional cartilage organoid systems, alongside emerging single-cell and spatial transcriptomic technologies, to better characterize cell-type-specific ncRNA regulatory networks in OA. Moreover, preclinical therapeutic studies require validation in larger animal models with longer intervention periods and functional outcome assessments. Because many OA-associated ncRNAs remain insufficiently characterized, comprehensive gain- and loss-of-function studies are needed to establish causal mechanisms. Continued advances in RNA biology and targeted delivery systems, together with insights from clinically approved RNA therapeutics, may ultimately accelerate the development of ncRNA-based precision diagnostics and disease-modifying therapies for OA.
